# Myricetin Exerts its Apoptotic Effects on MCF-7 Breast Cancer Cells through Evoking the BRCA1-GADD45 Pathway

**DOI:** 10.31557/APJCP.2020.21.12.3461

**Published:** 2020-12

**Authors:** Nayereh Sajedi, Mansour Homayoun, Fakhrossadat Mohammadi, Mitra Soleimani

**Affiliations:** 1 *Department of Anatomical Sciences, Isfahan University of Medical Sciences, Iran. *; 2 *Institute for Advanced Studies in Basic Sciences, Zanjan, Iran. *

**Keywords:** Myricetin, flavonoid, MCF-7, apoptosis

## Abstract

**Objective:**

Myricetin is a polyphenol flavonoid with nutraceutical values which is abundantly found as the main ingredient of various foods and beverages. It has been reported that the function of myricetin is to trigger apoptosis in several types of cancers. The present study intended to investigate the apoptotic effects of myricetin on MCF-7 breast cancer cells and to assess its possible mechanisms of action.

**Materials and Methods:**

MCF-7 breast cancer cells were assigned to four groups: Control (cells in normal condition); myricetin (cells treated with the IC50 dosage of myricetin) in three different incubation times (24, 48, and 72 h). The 3-(4,5-dimethylthiazol-2-yl)-2,5-diphenyltetrazolium bromide (MTT) assay, annexin V assay, flow cytometry, real-time polymerase chain reaction (PCR), and caspase-3 assay were used to estimate the apoptosis function of myricetin in breast cancer.

**Results:**

The expression levels of apoptosis-related genes *caspase-3, caspase-8, caspase-9*, and the *BAX /Bcl-2* ratio as well as the expression of *p53, BRCA1, GADD45* genes were significantly increased following the treatment of MCF-7 breast cancer cells with myricetin. The annexin V assay demonstrated the significant expression of annexin which was also detected by flow cytometry.

**Conclusion:**

Myricetin efficiently induces apoptosis in MCF-7 breast cancer cells by evoking both extrinsic and intrinsic apoptotic pathways. Myricetin may exert its apoptotic effects on MCF-7 cells by inducing the BRCA1- GADD45 pathway.

## Introduction

Breast cancer is the most frequently diagnosed cancer among women and the leading cause of cancer-related death among women worldwide (Torre et al., 2017). Natural supplements have long been suggested as remedies for preventing various types of cancer including breast cancer. However, using natural foods for the management of cancer (nutritional cancer therapy) is still in its early stages. Studies on the role of nutritional interventions during breast cancer have shown that supplementation with some dietary constituents enhances therapeutic efficacy. Accordingly, nutritional intervention in breast cancer is considered an integral part of the multimodal therapeutic approach (De Cicco et al., 2019). The epidemiological evidence demonstrates that the risk of certain cancers could be reduced with a diet rich in fruits and vegetables and this effect can mainly be attributed to natural polyphenols (Zhou et al., 2016). Polyphenols are widely present in plant-based foods and beverages. Natural polyphenols are divided into five classes including flavonoids, phenolic acids, lignans, stilbenes, and other polyphenols. Flavonoids and phenolic acids are the most common classes and account for about 60% and 30% of all natural polyphenols, respectively (Gutiérrez-Grijalva et al., 2017). Flavonoids are secondary plant metabolites that are responsible for the color and aroma of flowers. It has been suggested that interference of flavonoids in several signal transduction pathways in the process of carcinogenesis leads to a reduction in proliferation, angiogenesis, and metastasis (Ravishankar et al., 2013). 3,5,7-trihydroxy-2-(3,4,5-trihydroxyphenyl)-4-chromenone (myricetin) is a polyphenol flavonoid with nutraceutical values, which is abundantly found as the main ingredient of various herbal foods and beverages. Pathophysiological properties of myricetin, including its antioxidant, cytoprotective, antiviral, antimicrobial, and antiplatelet effects have been shown (Li et al., 2019). The anticancer properties of myricetin have also been reported by several studies. It has been shown that myricetin effectively represses the malignant progression of prostate cancer by inhibiting PIM1 and disrupting the PIM1/CXCR4 interaction (Ye et al., 2018); it also exerts potent anticancer effects on human skin tumor cells by inhibiting Fyn kinase activity and therefore attenuating UVB-induced COX-2 expression (Jung et al., 2008), inhibits angiogenesis by inducing reactive oxygen species (ROS)-mediated apoptosis and inhibiting the PI3K/Akt/mTOR signaling pathway (Kim, 2017), and suppresses the propagation of hepatocellular carcinoma by targeting YAP and its target genes (Li et al., 2019). However, the mechanism by which myricetin induces its apoptotic effects in breast cancer is not well understood. It has been demonstrated that myricetin suppresses p21-activated kinase 1 in human breast cancer MCF-7 cells through downstream signaling of the β-catenin pathway (Jiao and Zhang, 2016). Apoptosis is a coordinated process mediated by the activation of a group of cysteine proteases called caspases that exist as inactive zymogens or proenzymes. There are two main apoptotic pathways: the extrinsic or death receptor pathway and the intrinsic or mitochondrial pathway. Both pathways end with a final common effector pathway, known as the execution phase, and new pieces of evidence indicate that these two pathways are linked such that the molecules in one pathway can influence the other. To date, ten main caspases have been categorized as initiators (caspase-2, -8, -9, -10), effectors or executioners (caspase-3, -6, -7), and inflammatory caspases (caspase-1, -4, -5) (Elmore., 2007). The control and regulation of apoptotic mitochondrial events occur through the members of the Bcl-2 family of proteins (Adams and ,Cory 2007). It is thought that the main mechanism of action of the Bcl-2 family proteins is the regulation of cytochrome c release from mitochondria via the alteration of mitochondrial membrane permeability. To date, a total of 25 genes have been identified in the *Bcl-2* family. Some of the anti-apoptotic proteins include *Bcl-2, Bcl-x, Bcl-XL*, *Bcl-XS, Bcl-w,* and *BAG*, and some of the pro-apoptotic proteins include *Bcl-10, Bax, Bak, Bid, Bad, Bim, Bik,* and *Blk.* These proteins have special significance since they can determine if the cell commits to apoptosis or aborts the process (Wang and Youle, 2009). The tumor suppressor protein p53 has a critical role in the regulation of the Bcl-2 family of proteins (Hemann and Lowe, 2006). *p53* is almost the most commonly mutated or silenced gene in cancer, hence the most extensively studied one. Moreover, emerging evidence has assigned other important roles for p53 in the regulation of metabolism and cell homeostasis without causing cell cycle arrest or apoptosis (Liu et al., 2019). BRCA1 (BReast CAncer 1), which is located on chromosome 17, was the first studied gene involved in susceptibility to breast cancer. BRCA1 is a multifunctional protein that is involved in transcriptional activity, cell cycle regulation, and apoptosis (Minami et al., 2016) and more than 300 types of BRCA1 deletions have been identified to date (Gorski et al., 2009). Gadd45 is a p53-regulated growth arrest and DNA-damage-inducible gene that is also regulated in a p53-independent manner. It has recently been demonstrated that microinjection of the exogenous gadd45 into human fibroblasts induces G2 arrest but not apoptosis. Interestingly, microinjection of the p53 expression vector into the same cells did induce apoptosis, which highlights the specificity of gadd45’s function. The expression of gadd45 is up-regulated in a number of different cell types by genotoxic agents that are known to induce apoptosis (Sheikh et al., 2000). MCF-7 is a luminal A type, progesterone and estrogen receptor-positive breast cancer cell line. Since its development in 1973, the MCF-7 cell line has been commonly propagated and used by multiple research groups (Comşa et al., 2015). This study aimed to investigate the apoptotic effects of myricetin in the MCF-7 breast cancer cell line. Due to the important roles of the *p53, BCL2, BRCA1,* and *gadd45 *genes stated earlier, the changes in the expression of these genes in MCF-7 cells following treatment with myricetin were also evaluated. 

## Materials and Methods

*Cell culture*

MCF-7 breast cancer cells were purchased from the National Cell Bank, Pasteur Institute of Iran, Tehran. The cells were cultured in Dulbecco’s modified Eagle medium (DMEM)/F12 containing 10% fetal bovine serum (FBS) (Sigma, USA) supplemented with 1% penicillin and streptomycin (Sigma, USA) and incubated at 37°C, 95% humidity, and 5% CO_2_. At 80% confluency, the cells were trypsinized and incubated for the downstream experiments.

*3-(4,5-dimethylthiazol-2-yl)-2,5-diphenyltetrazolium bromide (MTT) assay and determining the IC50 dosage of myricetin*

Myricetin (Sigma, USA) was dissolved in dimethyl sulfoxide (DMSO) to achieve a 100 mmol/L stock solution and stored at -20^o^C. The IC_50_ dosage, the concentration of myricetin that inhibits half-maximal proliferation of MCF-7 cells (Yifeng, et al 2016), was determined as follows: 7,500 cells per well were seeded in 96-well plates and incubated overnight. Next, the cells were treated with a 200-µL serial dilution of myricetin (175, 125, 100, 75, 50, 25, and 10 μM) for 24 h. Afterwards, the cells underwent the MTT assay to determine the cell viability rate using a previously described protocol (Ahmadian et al., 2009). The assays included blank wells containing only the medium, untreated control cells, and test cells treated with myricetin in serial dilutions. Next, 50 μL of 2 mg/mL MTT (Sigma, USA) solution diluted in DMEM-F12 was added to each well. The plates were incubated at 37°C, 95% humidity, and 5% CO_2_ for 4 h. The medium was removed and 200 μL of DMSO was added to each well to dissolve the formazan. The wells were covered and agitated in an orbital shaker for 15 min. The absorbance in each well was measured at the wavelength of 570 nm in a microtiter plate reader. The reference wavelength was higher than 650 nm. The blanks were given values close to zero (+/- 0.1). Then the IC_50_ dosage curve was drawn. Based on the depicted curve, the concentration of 54 mM corresponded to 50% cell viability of MCF-7 cells following treatment with myricetin and was considered as the IC_50_ dosage of myricetin in this study.

*Experimental groups*

MCF-7 cells were assigned to four groups: The control group (cells in normal condition); and the myricetin groups, in which cells treated with 54 mM myricetin (the IC_50_ concentration) were divided into three groups in terms of the duration of treatment with myricetin (myr24, myr48, and myr72). A control group was designated for each time duration and accompanied it till the end of the experiment.

*Annexin V staining assay and flow cytometry*

During the early apoptosis, cell membrane asymmetry is rapidly lost without concomitant loss of membrane integrity. This results in the exposure of phosphatidylserine (PS) at the outer leaflet of the plasma membrane, which serves a physiological role in the recognition and subsequent removal of the dying cell by means of phagocytosis. Annexin V shows a high affinity for PS residues in the presence of millimolar concentrations of Ca^2+^. These apoptotic cells can be distinguished from annexin V-negative living cells using the flow cytometric procedure. By simultaneous probing of membrane integrity via staining with the nuclear dye propidium iodide (PI), apoptotic cells can be discriminated from necrotic cells (annexin V-: PI-) (Schutte et al., 1998). Annexin V- FITC apoptosis detection kit (eBioscience, #88-8005, USA) was used for the assay. Following 24, 48, and 72 h of treatment with 54 µM myricetin, the cells were trypsinized and washed with PBS. After adding the binding buffer, the cells were treated with 5 μL of annexin V-FITC. Next, the cells were incubated at room temperature for 15 min and then washed with washing buffer. Finally, 200 µL of buffer and 5 µL of PI were added to the cells and the apoptotic cells were counted via flow cytometry (Becton Dickinson, Heidelberg, Germany). The experiments were performed in triplicate independently and at three different times.

*Real-time polymerase chain reaction (PCR)*

The expression levels of *P53, BRCA1, GADD45, BAX, caspase-3, caspase-8, caspase-9*, and *Bcl-2* were determined by real-time PCR. The cells were treated with 54 mM myricetin for 24, 48, and 72 h. Total RNA in all the groups was extracted using the YTA Total RNA Purification Mini kit (Yekta Tajhiz Azma, Iran) according to the manufacturer’s protocol. After treatment with DNase I to remove the genomic DNA, cDNA was reverse transcribed using RevertAid™ First Strand cDNA Synthesis kit (Fermentas). Maxima SYBR Green ROX qPCR Master Mix kit (Fermentas) was used according to the manufacturer’s protocol in an ABI StepOnePlus™ Real-Time PCR System (Applied Biosystems). The cycling parameters were as follows: 10 min at 95°C for the initial denaturation followed by 40 cycles of the denaturation step at 95°C for 15 s and annealing/extension for 1 min at 60°C. β-actin was used as a reference gene for internal control. Data were analyzed using the comparative Ct (ΔΔct) method. The experiments were carried out in triplicate and were independently repeated at least three times. Gene-specific primer sequences are presented in Table 1. 

*Caspase-3 activity assay*

The caspase-3/CPP32 fluorometric assay kit (BioVision, Catalog, and K105-25) was utilized to evaluate the activity of caspase-3. A critical executioner of apoptosis, caspase-3 is responsible for the proteolytic cleavage of many key proteins. This assay is a fluorescence technique that detects the activity of caspase-3 in cell lysates. It contains a fluorogenic substrate (N-Acetyl-Asp-Glu-Val-Asp-7-amino-4-methylcoumarin or Ac-DEVD-AMC or -AFC) for caspase-3. During the assay, the activated caspase-3 cleaves this substrate between DEVD and AMC or AFC, generating a high fluorescence signal that can be detected using a fluorescence reader with the excitation wavelength at 380 nm and the emission wavelength at 420–460 nm. The more the number of apoptotic cells in the sample, the more the caspase-3 activity and the generation of fluorescence emission (Ponder and Boise 2019). MCF-7 cells were treated with 54 µM myricetin for 24, 48, and 72 h. Next, the cells were trypsinized and washed with PBS. The cell pellet was suspended in 50 µL of chilled cell lysis buffer. Then 50 µL of 2X reaction buffer (containing 10 mM dithiothreitol or DTT) was added to each sample. Next, the cells were incubated on ice for 10 min. Subsequently, 50 μL of 2X reaction buffer, 1 μL of DTT (1 M), and 5 μL of DEVD-AFC (1 mM) were added to the cell lysates. The reactions were incubated for 2 h at 37°C, 5% CO_2_, and 95% humidity. Finally, 50 μL of the cell lysates was transferred to a 96-well plate and the absorbance was determined using a fluorometric spectrophotometer with 400 excitation and 505-nm emission filters. DEVD-AFC emits blue light at 400 nm. 

*Statistical analysis*

To determine the statistically significant differences between the groups, one-way analysis of variance (ANOVA) along with Tukey’s post hoc test was performed using SPSS software package 25.0. The quantitative data are presented as mean ± standard deviation (SD). P-value <0.05 was considered as statistically significant. 

## Results

*Morphology of MCF-7 breast cancer cells*

**Figure 1:**
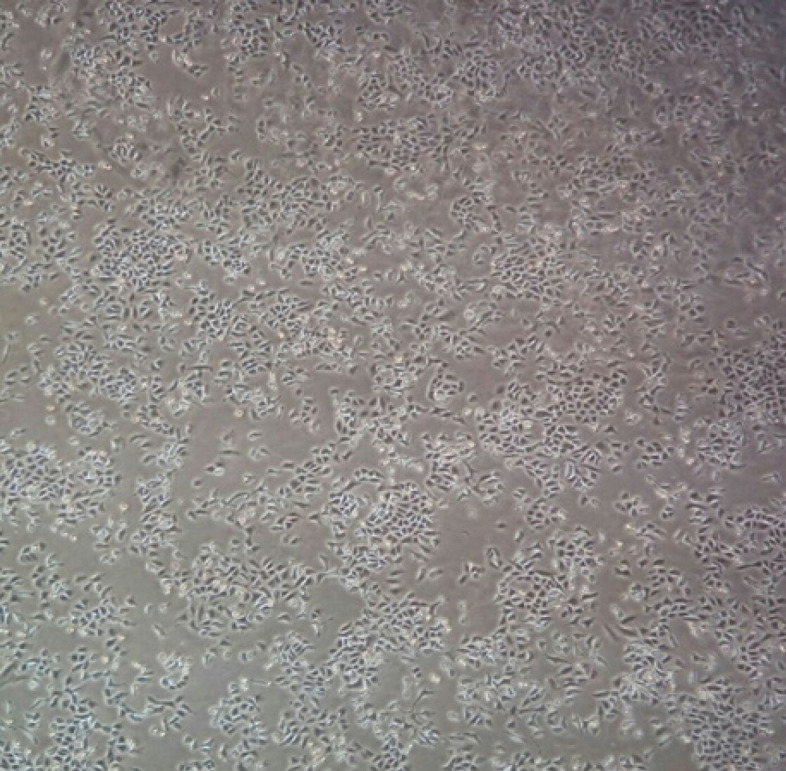
A Typical Cobblestone-Like Morphology of MCF-7 Breast Cancer Cells Displayed in Monolayer Culture, 40X

MCF-7 breast cancer cells displayed a typical cobblestone-like morphology in a monolayer culture (Figure 1).

*MTT assay and the IC*_50_ *dose-response curve*

**Figure 2:**
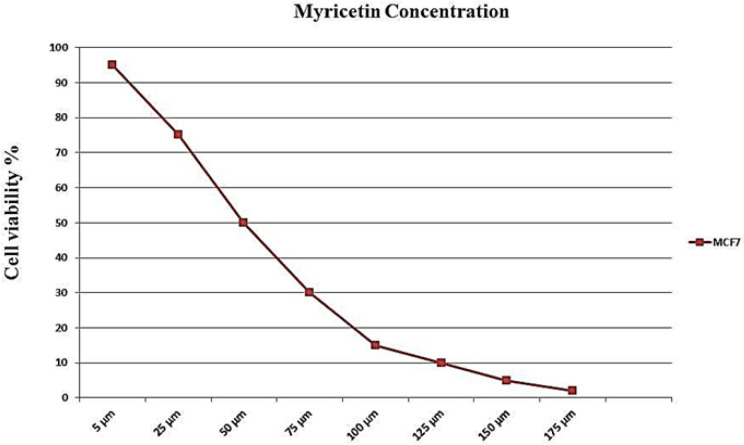
IC_50_ Assay for Half-Maximal Inhibitory Concentration Analysis of Myricetin in MCF-7 Cancer Cells after 24 hours of Treatment. Cells were treated with or without the myricetin in serial dilutions (175, 125, 100, 75, 50, 25 and10 μM), and the relative amount of viable cells were estimated by measuring the absorbance of the cell suspension after incubation. MTT was carried out and a dose response graph of viability versus myricetin concentration used to calculate IC50 values for MCF-7 cells. The graph pointed the concentration of 54 µM as IC50 for myricetin

Based on the results of the MTT assay, a dose-response curve was drawn (Figure 2). The point on the curve that corresponds to 50% viability coincided with a point in the x-axis of the curve (representing the concentration of myricetin), which is the IC_50_ dosage of myricetin.

*Treatment with the IC*_50_ *dosage of myrecitin and cell viability assay*

**Figure 3:**
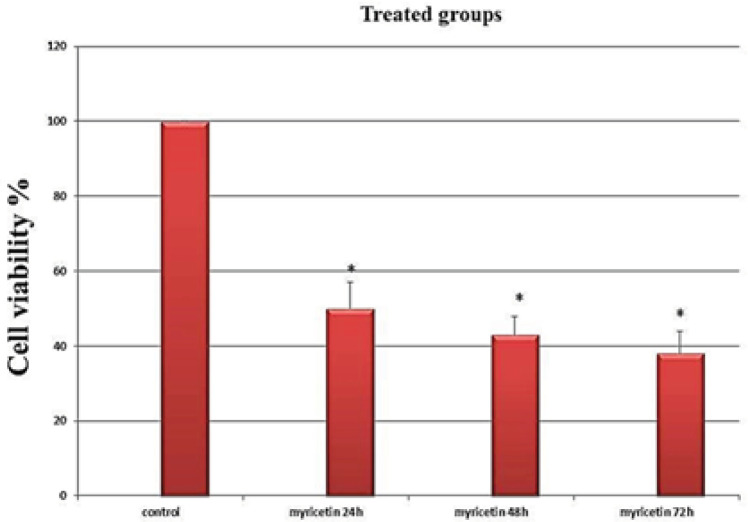
The Viability Assay of the MCF-7 Breast Cancer Cells Following Treatment with Myricetin as Evaluated by MTT Assay. Cells treated with half maximal inhibition concentration of myricetin (54µM) for 24, 48 and 72 hours. The viability of the cells decreased significantly with time. Data is represented as mean ± SD. * p< 0.01

MCF-7 cells were treated with 54 μM myricetin (the IC_50_ dosage). Cell viability was evaluated by the MTT assay. The results indicated that the viability of the cells significantly decreased following exposure to myricetin (p-value <0.01). These anti-proliferative effects increased incrementally with time (Figure 3).

*Annexin V assay and flow cytometry*

**Figure 4:**
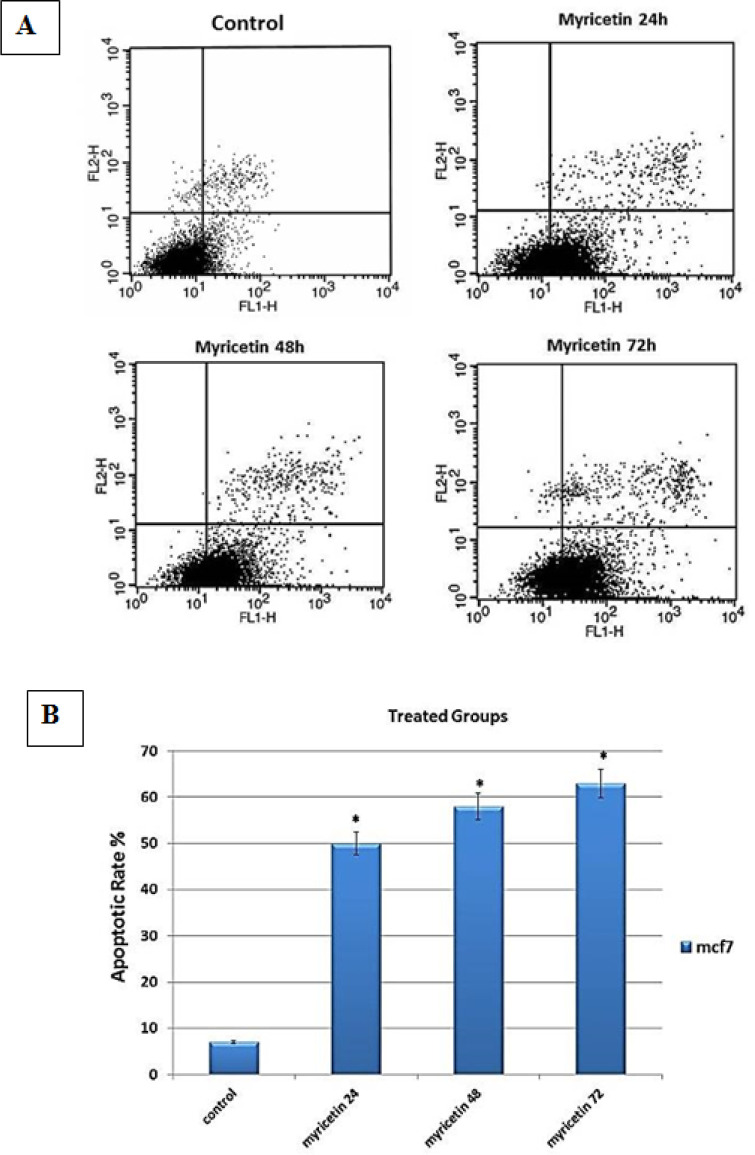
Flow Cytometric Analysis of Myricetin-Treated MCF-7 Cells. (A) The dot plot diagrams represent typical apoptotic and necrotic cell populations detected by annexin V-FITC and PI staining. The lower left quadrants of the panels show viable intact cells, which were negative for annexin V-FITC binding and excluded PI (FITC-/PI-); the upper right quadrants show late apoptotic cells, which were positive for annexin V-FITC binding and PI uptake (FITC+/PI+). The lower right quadrants represent early apoptotic cells, positive for annexin V-FITC and negative for PI staining (FITC+/PI-). (B) The bar graph shows the average percentage of apoptotic cells. The values are presented as means ± SD. *p<0.01

Flow cytometry was performed to determine the rate of annexin V-positive cells. The results showed a significant increase in the apoptosis rate in MCF-7 cells treated with 54 μM myricetin (the IC_50_ dosage) (p-value <0.01) (Figure 4).

*Real-time PCR*

**Figure 5:**
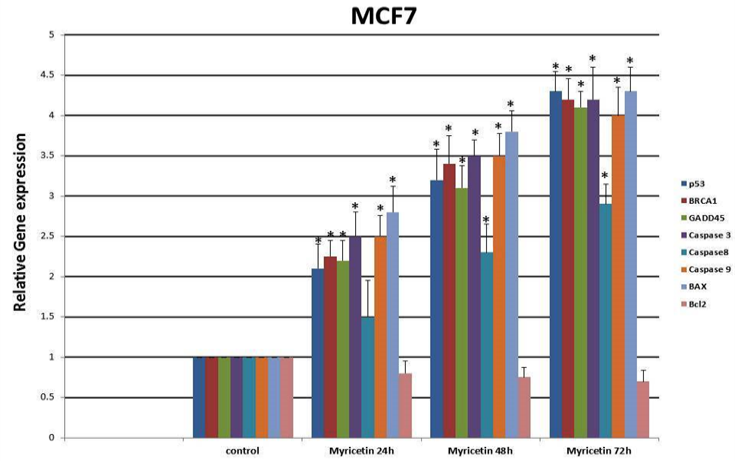
Real-Time PCR Gene Expression Analysis of MCF-7 Cells Treated by 54 µm Myricetin for 24, 48, and 72 hrs. The expression of P53, BRCA1, GADD45, Caspase-3, Caspase-8, Caspase-9 and BAX increased and the expression of the Bcl2 gene decreased significantly by time compared to control group. Data are represented as mean ± SD. *p<0.01

The expression levels of *P53, BRCA1, GADD45, BAX, caspase-3, caspase-8, caspase-9, *and *Bcl-2* were determined by real-time PCR. The expression of *p53, BAX, GADD45, BRCA1, caspase-3, caspase-8 *and *caspase-9 *genes increased while the expression of Bcl-2 decreased significantly with time compared with the control group (Figure 5).

*Caspase-3 activity assay*

**Figure 6:**
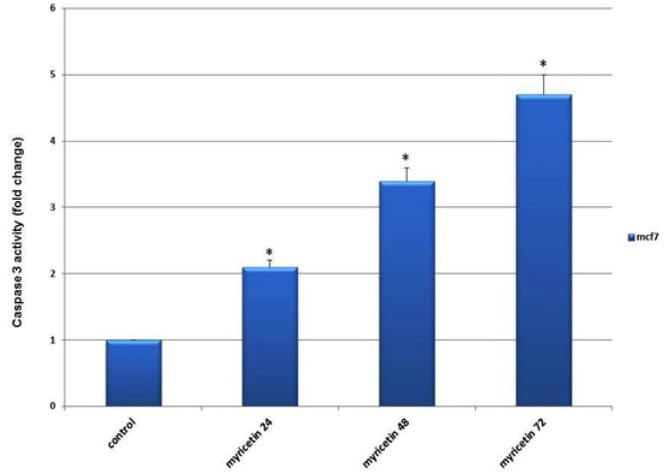
The Caspase-3 Activity in MCF-7 Breast Cancer Cells Plotted against duration of Exposing Cells to 54 µm Myricetin (24, 48 and 72 hrs). The Caspase-3 activity increased with time significantly compared to control group. The data is expressed as the percentage of cells that show a cytoplasmic fluorescence intensity greater than 450 (Z’= 0.51)

Caspase-3 activity in breast cancer MCF-7 cells was measured to ensure cell death via apoptosis. The results showed a significant increase in caspase-3 activity in the myricetin-treated groups compared with the control group. Caspase-3 activity augmented up to 72 h after treatment with myricetin (Figure 6).

**Table 1:** Primer Sequences Used in the Real-Time PCR

Target	Primer Sequences
Bcl-2	F: AAAATACAACATCACAGAGGAAG
	R: CTTGATTCTGGTGTTTCCC
Caspase 9	F: CCTTTGTTCATCTCCTGCTTAG
	R: CCTCAAACTCTCAAGAGCACC
BRCA1	F: TGTTACAAATCACCCCTCAAG
	R: CCTGATACTTTTCTGGATGCC
GADD45	F: TTTTGCTGCGAGAACGAC
	R: GAACCCATTGATCCATGTAG
Caspase 3	F: AGCACTGGAATGACATCTCG
	R: ACATCACGCATCAATTCCAC
TP53	F: CACTCCAGCCACCTGAAGTC
	R: GCAAGCAAGGGTTCAAAGAC
BAX	F: GGAGCTGCAGAGGATGATTG
	R: GTCCAATGTCCAGCCCATG
Caspase 8	F: ACTGGATGATGACATGAACCTG
	R: GCTGAATTCTTCATAGTCGTTG
Beta actin	F: TTCGAGCAAGAGATGGCCA
	R: CACAGGACTCCATGCCCAG

## Discussion

In this study, the apoptotic effects of myricetin, an herbal polyphenol flavonoid, on MCF-7 breast cancer cells were investigated. The results demonstrated that myricetin efficiently and extensively induces apoptosis in MCF-7 breast cancer cells. Several studies have reported the apoptotic effects of myricetin in cancer (Kim et al., 2014; Seydi, 2016). To examine the apoptotic function of myricetin on MCF-7 cells, the expression of annexin V, a protein with high affinity to PS in a calcium-dependent manner (Schutte et al., 1998), was assessed and the results demonstrated that the IC_50_ dosage of myricetin efficiently inhibited the growth of the cells. To assess the apoptosis caspase pathways in this study, *caspase-3*, *-8,* and *-9* gene expressions were evaluated. *Caspase-8* and *-9* are the essential initiator caspases required for apoptosis signaling through the mitochondrial pathway (the intrinsic pathway). Caspase-3, an executioner caspase, is activated in both extrinsic and mitochondrial apoptosis pathways and plays an important role in the extrinsic activation of the apoptosis pathway. To start its activity, caspase-3 zymogen should be cleaved by caspase-8 and -9 (McIlwain et al., 2015). Overexpression of the *caspase-3, -8,* and *-9* genes in the current study as well as the extensive expression of caspase-3 protein, as confirmed by the caspase-3 assay, demonstrates that myricetin exerts its apoptotic effect on MCF-7 breast cancer cells by evoking both intrinsic and extrinsic pathways. The mitochondrial (intrinsic) apoptotic pathway is mainly regulated through members of the Bcl-2 family of proteins. They regulate apoptosis by either anti-apoptotic or pro-apoptotic functions (Adams and Cory, 2007). The *Bcl2* gene belongs to the former group and BAX belongs to the latter. Increasing the BAX/BCl2 ratio is an important factor for the induction and processing of apoptosis in breast cancer (Azimian et al., 2018). Previous experiments have reported a significant rise in the BAX/BCl2 ratio following the administration of myricetin in human hepatocarcinoma (Li et al., 2019) and human colon cancer (Kim et al., 2014). In the present investigation, the *BAX* gene was significantly overexpressed, while the expression of Bcl2 decreased following the treatment of MCF-7 cells with myricetin, which led to an increase in the BAX/Bcl2 ratio. This finding was in agreement with the results previously mentioned. The current study also investigated the alterations in the expression of tumor suppressor genes *p53* and *BRCA1*, two fundamental genes in the DNA damage response pathways that have compensatory roles in DNA repair (Hartman and Ford 2003) and their mutations leads to predisposition to a variety of cancers including and particularly, breast cancer. It has been demonstrated that p53 is required to modulate the expression of BRCA1 (Arizti et al., 2000). It also has been suggested that mutated p53 and BRCA1 collaborate to evolve malignant breast cancer (triple negative) (Kumar et al., 2012). The association between BRCA1 and p53 status in breast cancer has also been elucidated through a meta-analysis study (Peng et al., 2016). Moreover, it has been shown that BRCA1 can regulate p53-dependent transcription (MacLachlan et al., 2002). Studies have reported that overexpression of BARD1, a BRCA1-associated protein, leads to cell death with all the features of apoptosis (Irminger-Finger et al., 2001). In the present study, the expression of p53 and BRCA1 augmented incrementally after exposure to myricetin. To further investigate the apoptotic effects of myricetin in breast cancer, the expression of GADD45, a central target in tumorigenesis, was evaluated in this study. Functional evidence has indicated that GADD45 acts as a tumor suppressor and its defects are associated with the initiation of malignancies. GADD45 is a p53-regulated and stress-inducible gene (Tamura et al., 2012). Furthermore, it has been suggested that overexpression of BRCA1 induces the expression of GADD45 by activating its promoter (Jin et al., 2000). Overexpression of GADD45 in the present study, as evaluated by gene expression analysis, is another proof for the apoptosis inducing effect of myricetin.

In conclusion, based on the findings of this study, it can be concluded that: 1) myricetin efficiently induces apoptosis in MCF-7 breast cancer cells; 2) myricetin meddles with both extrinsic and intrinsic apoptotic pathways; and 3) myricetin may exert its apoptotic effects on MCF-7 cells via inducing the BRCA1-GADD45 pathway.

## References

[B1] Adams JM, Cory S (2007). The Bcl-2 apoptotic switch in cancer development and therapy. Oncogene.

[B2] Ahmadian S, Barar J, Saei AA, Fakhree MA, Omidi Y (2009). Cellular toxicity of nanogenomedicine in MCF-7 cell line: MTT assay. J Vis Exp.

[B3] Arizti P, Fang L, Park I (2000). Tumor suppressor p53 is required to modulate BRCA1 expression. Mol Cell Biol.

[B4] Azimian H, Dayyani M, Toossi MTB, Mahmoudi M (2018). Bax/Bcl-2 expression ratio in prediction of response to breast cancer radiotherapy. Iran J Basic Med Sci.

[B5] Comşa Ş, Cîmpean AM, Raica M (2015). The story of MCF-7 breast cancer cell line: 40 years of Experience in Research. Anticancer Res.

[B6] De Cicco P, Catani MV, Gasperi V2 (2019). Nutrition and Breast Cancer: A Literature Review on Prevention, Treatment and Recurrence. Nutrients.

[B7] Elmore S (2007). Apoptosis: a review of programmed cell death. Toxicol Pathol.

[B8] Gorski JJ, Kennedy RD, Hosey AM, Harkin DP (2009). The complex relationship between BRCA1 and ERalpha in hereditary breast cancer. Clin Cancer Res.

[B9] Gutiérrez-Grijalva EP, Picos-Salas MA, Leyva-López N (2017). Flavonoids and phenolic acids from Oregano: occurrence, biological activity and health benefits. Plants (Basel).

[B10] Hartman AR, Ford JM (2003). BRCA1 and p53: compensatory roles in DNA repair. J Mol Med.

[B11] Hemann MT, Lowe SW (2006). The p53–Bcl-2 connection. Cell Death Differ.

[B12] Irminger-Finger I, Leung WC, Li J (2001). Identification of BARD1 as mediator between proapoptotic stress and p53-dependent apoptosis. Mol Cell.

[B13] Jiao D, Zhang XD (2016). Myricetin suppresses p21-activated kinase 1 in human breast cancer MCF-7 cells through downstream signaling of the β-catenin pathway. Oncol Rep.

[B14] Jin S, Zhao H, Fan F (2000). BRCA1 activation of the GADD45 promoter. Oncogene.

[B15] Jung SK, Lee KW, Byun S (2008). Myricetin suppresses UVB-induced skin cancer by targeting Fyn. Cancer Res.

[B16] Kim GD (2017). Myricetin inhibits angiogenesis by inducing apoptosis and suppressing PI3K/Akt/mTOR signaling in endothelial cells. J Cancer Prev.

[B17] Kim ME, Ha TK, Yoon JH, Lee JS (2014). Myricetin induces cell death of human colon cancer cells via BAX/BCL2-dependent pathway. Anticancer Res.

[B18] Kumar P, Mukherjee M, Johnson JP (2012). Cooperativity of Rb, Brca1, and p53 in malignant breast cancer evolution. PLoS Genet.

[B19] Li M, Chen J, Yu X (2019). Myricetin suppresses the propagation of hepatocellular carcinoma via down-regulating expression of YAP. Cell.

[B20] Liu J, Zhang C, Hu W, Feng Z (2019). Tumor suppressor p53 and metabolism. J Mol Cell Biol.

[B21] MacLachlan TK, Takimoto R, El-Deiry WS (2002). BRCA1 directs a selective p53-dependent transcriptional response towards growth arrest and DNA repair targets. Mol Cell Biol.

[B22] McIlwain DR, Berger T, Mak TW (2015). Caspase functions in cell death and disease. Cold Spring Harb Perspect Biol.

[B23] Minami A, Murai T, Nakanishi A (2016). Cell cycle regulation via the p53, PTEN, and BRCA1 tumor suppressors. Chapter.

[B24] Peng L, Xu T, Long T, Zuo H (2016). Association between BRCA status and P53 status in breast cancer: A Meta-Analysis. Med Sci Monit.

[B25] Ponder KG, Boise LH (2019). The prodomain of caspase-3 regulates its own removal and caspase activation. Cell Death Discov.

[B26] Ravishankar D, Rajora AK, Greco F, Osborn HM (2013). Flavonoids as prospective compounds for anti-cancer therapy. Int J Biochem Cell Biol.

[B27] Schutte B, Nuydens R, Geerts H, Ramaekers F (1998). Annexin V binding assay as a tool to measure apoptosis in differentiated neuronal cells. J Neurosci Methods.

[B28] Seydi E, Rasekh HR, Salimi A, Mohsenifar Z, Pourahmad J (2016). Myricetin selectively induces apoptosis on cancerous hepatocytes by directly targeting their mitochondria. Basic Clin Pharmacol Toxicol.

[B29] Sheikh MS, Hollander MC, Fornance AJ Jr (2000). Role of Gadd45 in apoptosis. Biochem Pharmacol.

[B30] Tamura RE, de Vasconcellos JF, Sarkar D 2012). GADD45 proteins: central players in tumorigenesis. Curr Mol Med.

[B31] Torre LA, Islami F, Siegel RL, Ward EM, Jemal A (2017). Global cancer in women: Burden and Trends. Cancer Epidemiol Biomarkers Prev.

[B32] Wang C, Youle RJ (2009). The role of mitochondria in apoptosis. Annu Rev Genet.

[B33] Yifeng He, Qiujing Zhu, Mo Chen (2016). The changing 50% inhibitory concentration (IC50) of cisplatin: a pilot study on the artifacts of the MTT assay and the precise measurement of density-dependent chemoresistance in ovarian cancer. Oncotarget.

[B34] Ye C, Zhang C, Huang H (2018). The natural compound myricetin effectively represses the malignant progression of prostate cancer by inhibiting PIM1 and disrupting the PIM1/CXCR4 interaction. Cell Physiol Biochem.

[B35] Zhou Y, Zheng J, Li Y (2016). Natural polyphenols for prevention and treatment of cancer. Nutrients.

